# Constructing enriched stable consortia for enhanced fermentation of cigar tobacco leaves

**DOI:** 10.3389/fmicb.2026.1769511

**Published:** 2026-04-14

**Authors:** Tianfei Zheng, Dongfeng Guo, Yaqi Shi, Cunyong Zhang, Jinlong Zhou, Kun Zong, Guiming Hua, Xingjiang Li

**Affiliations:** 1 Technology Center, China Tobacco Anhui Industrial Co., Ltd., Hefei, Anhui, China; 2 School of Food and Biological Engineering, Hefei University of Technology, Anhui, Hefei, China

**Keywords:** cigar tobacco leaves, enriched microbial consortium, fermentation, high-throughput sequencing, sensory evaluation, top-down enrichment

## Abstract

**Introduction:**

Enriched microbial consortia have been proven to effectively regulate agricultural fermentation, yet their practical applications in the fermentation of cigar tobacco leaves are still limited.

**Methods:**

In this study, three natural microbial communities collected from high-quality cigar tobacco leaves were continuously cultured in a medium made of ordinary cigar tobacco leaves to construct enriched stable consortia via the top-down approach. Then, these constructed microbiotas were inoculated into ordinary cigar tobacco leaves to test their fermentation performance.

**Results:**

High-throughput sequencing results indicated that after three rounds of transfer culture, three simple and stable bacterial communities were formed, dominated by *Corynebacterium*, *Georgia*, *Bacillus* and *Enterococcus*. Fermentation with the enriched consortia significantly improved the aroma quality, aroma intensity, sweetness, and aroma richness of CTLs.

**Discussion:**

This study presented a straightforward method for constructing efficient and stable microbiotas based on microbial interactions and the material properties of cigar tobacco leaves in practical production, simplifying the development of microbial consortia for CTL fermentation.

## 1 Introduction

The microbial community in cigar tobacco leaves is crucial in the fermentation process (Zhang et al., 2024; Zheng et al., 2022a). Different from other solid-state fermentations with large-scale inoculation, the microbial community in cigar tobacco leaves stems from the uncontrolled external migration of microorganisms in the natural environment (Ren et al., 2023). As the fermentation of cigar tobacco leaves completely depends on the random migration of microorganisms in the natural environment, this dependence may lead to the instability of the fermentation effect and the quality difference between different batches. Inspired by the traditional fermentation methods of Chinese Baijiu and sourdough, inoculating a starter before fermentation might solve this problem (Du et al., 2020; Jin et al., 2017). Fermentation starter is a type of microbial agent for initiating or promoting fermentation (Rukwishuro et al., 2025).

Existing studies on cigar tobacco fermentation microorganisms mostly focus on the screening and application of single functional strains (e.g., *Bacillus*, *Staphylococcus*) (Liu et al., 2025; Yao et al., 2025). However, the natural environment contains abundant native microbial communities that can significantly affect the effectiveness and behavior of external strains (Zheng et al., 2022b). The mixed microbial agents (starter) contain multiple species with high functional and genetic diversity (Hakalehto et al., 2025). The synergistic interactions within the consortium, such as cross feeding, have shown promising potential in promoting their adaptation to the environment and multi-subject transformation (Niu et al., 2022).

The preparation of mixed microbial agents (starter) usually adopts bottom-up and top-down approaches (Gao et al., 2023; Jin et al., 2024). The bottom-up approach is based on the study of microbial individuals and communities (Peng et al., 2025). According to the metabolic characteristics of microbial individuals and the interactions between microorganisms, it synthesizes microbial communities with ideal functions (Du et al., 2023). Researchers have proposed using synthetic microbial communities with clear compositions and functions to prepare fermentation starters. Several groups have demonstrated that synthetic communities can be engineered to perform functions such as biodegradation of environmental contaminants, manipulation of plant phenotypes, or biofuel production (Afrizal et al., 2022; Chen et al., 2024; Gao et al., 2022). Despite these success stories, engineering consortia from the bottom-up remains challenging. First, effective screening systems and real-time detection systems are required to efficiently isolate and cultivate target microorganisms. The function of microbial communities is typically influenced by microbial interactions (Pierce and Dutton, 2022; Srinivasan et al., 2024). These interactions are difficult to predict based on first principles and expand rapidly as species richness increases (Oña et al., 2025). Due to the limited understanding of microbial communities, most can only construct simple communities with a restricted species composition. More importantly, microbial communities are rapidly evolving ecological systems (Kehila et al., 2025). The engineered functions can be disrupted by environmental fluctuations, invasive species, species extinctions or the fixation of mutant genotypes (Pignon and Schaerli, 2025). The top-down approach starts from the existing complex microbial communities in the natural environment (Chen et al., 2025). It simplifies, modifies, and optimizes these communities to obtain enriched stable consortia with specific functions (Gilmore et al., 2019). Top-down enrichment and domestication is an important method of microbial consortium engineering. Compared with bottom-up synthetic consortia, it has the advantage of making full use of the functional diversity and synergistic effects of natural microbial communities without in-depth understanding of the interaction mechanisms within the consortium, which is more suitable for solving complex problems in practical production (Gilmore et al., 2019; Chen et al., 2025).

**Figure 1:**
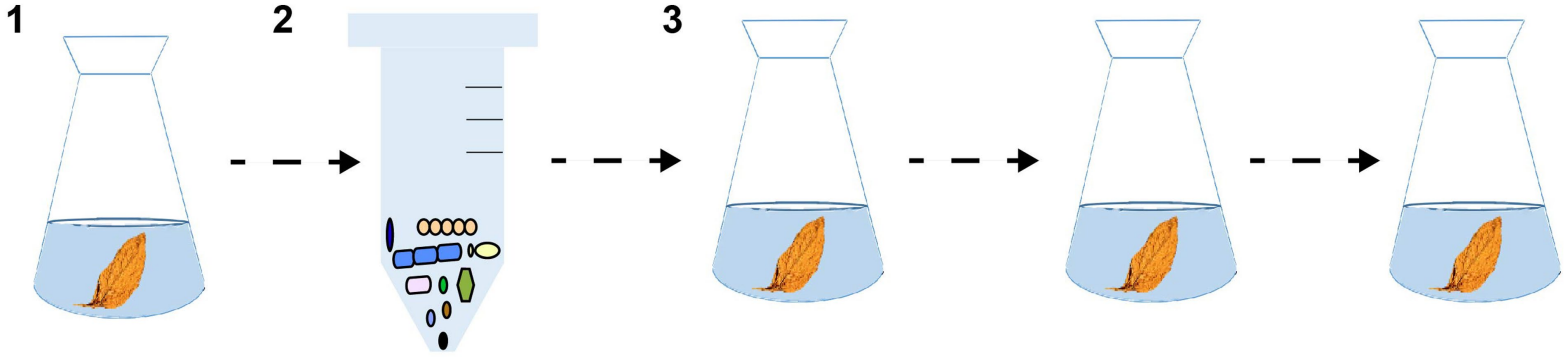
The construction process of enriched stable consortia. First, the natural microbial communities were enriched and isolated from high-quality cigar tobacco leaves. Subsequently, the natural microbial communities were continuously sub-cultured using the medium prepared by sterilizing ordinary cigar tobacco leaves. Under the effect of microbial interactions and nutritional components, the microbiotas with short-term laboratory stability would eventually form.

In this study, we constructed enriched stable consortia via the top-down approach (Figure 1). First, the natural microbial communities were enriched and isolated from high-quality cigar tobacco leaves. Subsequently, the natural microbial communities were continuously sub-cultured using the medium prepared by sterilizing ordinary cigar tobacco leaves. Under the effect of microbial interactions and nutritional components, the stable microbiotas would eventually form. Finally, the stable microbiotas were inoculated into cigar tobacco leaves for solid-state fermentation to test their fermentation performance. The specific research objective of this study is to employ natural microbial communities from high-quality cigar tobacco leaves as the foundational material, aiming to construct enriched and stable microbial consortia tailored for cigar tobacco fermentation through a top-down enrichment and domestication approach. This involves elucidating the succession dynamics, dominant genus composition, and microbial interactions within these enriched consortia. Furthermore, the study seeks to verify the enhancing effect of these enriched consortia on the fermentation quality of cigar tobacco leaves. Ultimately, it aims to establish a straightforward, efficient, and practical methodology for constructing microbial consortia for cigar tobacco fermentation, thereby offering a microbial-based solution to address the issue of inconsistent natural fermentation quality in cigar tobacco leaves.

## 2 Materials and methods

### 2.1 Materials

Three kinds of cigar tobacco leaves from Dominica, Indonesia, and Brazil were selected as raw materials to collect the natural microbial communities, and labeled as A, B, and C. The cigar tobacco leaves from Yunnan were used as raw materials for preparing culture media and validating the enriched stable consortia, and labeled as CK. When preparing the medium, 1 g cigar tobacco leaves powder (CK) was added to 100 mL of distilled water and sterilized at 115 °C for 20 min. This tobacco leaf medium is a pure cigar tobacco leaf matrix medium without adding any additional carbon sources, nitrogen sources or inorganic salts, aiming to simulate the natural nutritional environment of cigar tobacco leaves and realize the directional enrichment of adaptive consortia. To ensure the batch consistency of the medium, CK cigar tobacco leaves were collected from the same production area and the same maturity in Yunnan, mixed and ground to pass a 40-mesh sieve, and packaged as 10 g per portion for sealed storage. The same batch of tobacco leaf powder was used for each medium preparation, and the ratio of 1 g/100 mL was strictly followed, with the same sterilization condition of 115 °C for 20 min to ensure the consistency of nutrient components and physical and chemical properties of the medium in different batches.

### 2.2 Microbial community construction

The construction process of the enriched stable microbial consortium was shown in Figure 1. The natural microbial community on cigar tobacco leaves were collected using water washing method. Briefly, 5 g cigar tobacco leaves and 100 mL sterile normal saline (0.85% NaCl) were placed in a 250 mL flask, placed in a shaking table, shaken and eluted at 220 rpm for 3 h, filtered tobacco leaf fragments, and centrifuged at 7,000 × *g* for 15 min to collect microbial community. This method mainly obtains the loosely attached microbial community on the tobacco leaf surface. Since the core microorganisms of cigar tobacco leaf fermentation are all surface-colonizing microorganisms, the content of microorganisms inside the tobacco leaf is extremely low and difficult to participate in the fermentation process (Ren et al., 2023). Therefore, the consortium obtained by this method can truly reflect the functional microbial community of cigar tobacco leaf fermentation, and the bias is negligible. The collected microbial community was inoculated to the culture medium with an inoculum amount of 1% (v/v), and cultured at 30 °C and 220 rpm. Successive subcultures were performed after 5 days with the same inoculum amount. After three rounds of subcultures, the microbial community structure stabilized (Bray–Curtis dissimilarity <0.1 between the 2nd and 3rd generations, calculated by QIIME 2 with a rarefaction depth of 30,000 sequences). The natural microbial community was numbered as A0, B0 and C0, the successive cultured microbial community was numbered sequentially as A1, A2, A3, B1, B2, B3, C1, C2, and C3. Three biological replicates were set for each subculture sample for subsequent high-throughput sequencing analysis.

### 2.3 Determination of conventional chemical components in cigar tobacco leaves

Conventional chemical components in cigar tobacco leaves, including total nitrogen, total sugar, reducing sugar, and nicotine, were measured by continuous flow analysis based on tobacco industry standards YC/T61–2002, YC/T159–2019, YC/T217–2007, and YC/T468–2013.

### 2.4 Microbial community analysis

To track community succession during microbial community construction, the consortia were analyzed by amplicon sequencing. The suspension of the fermentation broth was filtered through sterile gauze and centrifuged at 7,000 × *g* for 15 min to collected microbial communities. Total genomic DNA of microbial communities was extracted using the DNeasy PowerSoil Kit (QIAGEN) according to the manufacturer’s protocol. Three technical replicates of DNA extraction were performed for each biological replicate of the sample, and the extracted DNA was mixed in equal amounts for subsequent PCR amplification and sequencing to avoid technical errors. DNA quality and concentration were assessed using a NanoDrop ND-1000 spectrophotometer (Thermo Fisher Scientific) and agarose gel electrophoresis. The bacterial and archaeal 16S rRNA genes V4–V5 region were amplified using the forward primer 515F (5′-GTGCCAGCMGCCGCGGTAA-3′) and the reverse primer 907R (5′-CCGTCAATTCMTTTRAGTTT-3′). The amplified PCR products were purified and quantified. Purified amplicons were sequenced on an Illumina MiSeq platform (2 × 300 bp chemistry) following standard protocols. Raw sequences were processed in QIIME 2 (Bolyen et al., 2019) through quality filtering of raw reads, denoising with DADA2, removal of chimeric sequences, and clustering into amplicon sequence variants (ASVs), with ASV filtering criteria set as follows: remove ASVs with a relative abundance of < 0.01% in all samples, and remove unclassified ASVs at the phylum level, resulting in a total of 1,245 valid ASVs for subsequent taxonomic and diversity analysis. Taxonomic annotation of ASVs was performed against the SILVA 138 database with a confidence threshold of 0.7; alpha diversity (Shannon index) was calculated to evaluate the species diversity of the microbial community, while beta diversity (weighted UniFrac distance) was used to analyze the structural differences of microbial communities with principal coordinate analysis (PCoA) for visualization. The rarefaction depth for all sequencing samples was uniformly set to 30,000 sequences per sample, and the rarefaction curves of all samples reached saturation with an average sequencing coverage of 99.2 ± 0.3%, indicating that the sequencing data could fully reflect the actual microbial community structure of the samples.

### 2.5 Validation of fermentation efficiency of the constructed synthetic microbial community

The stable enriched consortia (A3, B3, and C3) were inoculated into ordinary CK CTLs for solid-state fermentation to evaluate their fermentation performance, with each treatment group (A3/B3/C3) and control group set with three biological replicates, each containing 50 g of CTLs to ensure the statistical reliability of the results. For the fermentation validation experiment, an uninoculated control group (designated as CK-S) was established, where CK tobacco leaves underwent spontaneous fermentation under the same environmental conditions as the inoculated groups. Briefly, the stable consortia were cultured in the CTL-based medium for 5 days, and the culture broth was diluted 5 times with sterile water; the colony forming units (CFU) of the diluted inoculum were determined by the plate counting method (LB solid medium, 30 °C, 48 h) and controlled at (2.5 ± 0.3) × 10^8^ CFU/mL, with a consistent inoculum concentration for all treatment groups. The diluted inoculum was evenly sprayed on the CTLs with an inoculum amount of 30% (v/g); after inoculation, the exact moisture content of the CTLs was controlled at 35 ± 1% (determined by the oven drying method at 105 °C to constant weight), and the moisture content was maintained by supplementing sterile water every 3 days during fermentation. The inoculated CTLs were placed in a biochemical incubator and fermented at 30 °C and 75% relative humidity for 15 days, after which the CTLs were dried to a moisture content of 12 ± 1% at 45 °C and subjected to sensory evaluation. It should be noted that sterilized substrate and heat-killed inoculum controls were not included in this experiment due to practical production scenario simulation considerations, which is a limitation for direct causal inference of the microbial consortia’s biological activity on fermentation effects.

### 2.6 Sensory evaluation

Sensory evaluation serves as the ultimate basis for assessing the quality of cigar tobacco leaves (Krüsemann et al., 2019). The sensory evaluation experiments were independently repeated 3 times for all treatment and control groups; for each repetition, 10 cigars were hand-rolled for each group (50 mm in length, 10 mm in diameter) to ensure sufficient sampling, and the cigars were placed in a constant temperature and humidity environment (20 °C, 68 ± 1% relative humidity) for 48 h to balance the moisture before evaluation. A strict double-blind protocol was adopted for all sensory evaluation experiments to eliminate subjective bias: fermented CTLs of different groups were coded with random 3-digit numbers (excluding any group information), and cigars were rolled by professional technicians who were not involved in the fermentation experiment to avoid code exposure; the tasting panel was only provided with coded cigars and a unified quantitative scoring table, without any information about the treatment/control groups; tasting was conducted in a standard tobacco sensory evaluation room with constant temperature (20 °C), constant humidity (68 ± 1% RH), no external odor interference, and independent tasting positions for each panel member to avoid mutual communication. The tasting panel consisted of five professional tasters with formal tobacco industry cigar sensory evaluation qualification certification and 10–20 years of professional CTL sensory evaluation experience, and a 100-point quantitative scoring system was established for the sensory evaluation of CTLs, covering aroma quality [30 points, evaluating the purity, fineness and typicality of CTL aroma with scoring ranges of no peculiar smell (25–30), slight peculiar smell (20–24), obvious peculiar smell (15–19), severe peculiar smell (<15)], aroma intensity [20 points, evaluating the concentration and persistence of CTL aroma with scoring ranges of strong and persistent (17–20), moderate (13–16), weak (9–12), extremely weak (<9)], irritation [15 points, evaluating the stimulating sensation of smoke on the oral cavity, throat and nasal cavity with scoring ranges of no irritation (13–15), slight irritation (10–12), obvious irritation (7–9), severe irritation (<7)], aroma richness [20 points, evaluating the variety and layering of CTL aroma components with scoring ranges of rich and multi-layered (17–20), relatively rich (13–16), single (9–12), extremely single (<9)], and sweetness [15 points, evaluating the sweet taste of smoke in the oral cavity and sweet aftertaste with scoring ranges of strong sweet taste and obvious aftertaste (13–15), slight sweet taste (10–12), no sweet taste (7–9), bitter taste (<7)]. Each taster scored the cigars independently according to the above criteria; the final sensory score of each group was the average value of 3 independent repetitions (15 sets of scoring data in total), including the score of each single indicator and the total sensory score (sum of all single indicator scores).

### 2.7 Statistical analysis

R v. 4.0.0 was utilized to generate the heatmap, and principal component analysis (PCA), boxplot analysis, and multiple comparisons were performed. Shannon index was used for alpha diversity analysis for its advantage of simultaneously reflecting species richness and evenness; weighted UniFrac distance was used for PCoA analysis for its consideration of the evolutionary relationship between species, and PERMANOVA test with 999 permutations was used to verify the significance of community structure differences. Additionally, based on Spearman correlation coefficients (*p* < 0.05, |*r*| > 0.7), the correlation between the dominant microbes was analyzed, and network analysis was carried out using Gephi software. For all multiple comparison analyses (microbial community relative abundance, functional potential, chemical components, sensory scores), the Benjamini-Hochberg false discovery rate (FDR) correction was used for multiple testing correction, with the corrected *p* < 0.05 as the threshold for significant difference and *p* < 0.01 as the threshold for extremely significant difference; Cohen’s *d* effect size was calculated to evaluate the magnitude of the treatment effect, with Cohen’s *d* > 0.8 defined as a large effect size. All data were standardized during the statistical analysis, and all statistical analyses were based on the results of biological replicates, with data expressed as mean ± standard deviation (mean ± SD).

## 3 Results

### 3.1 Conventional chemical components in cigar tobacco leaves

**Figure 2:**
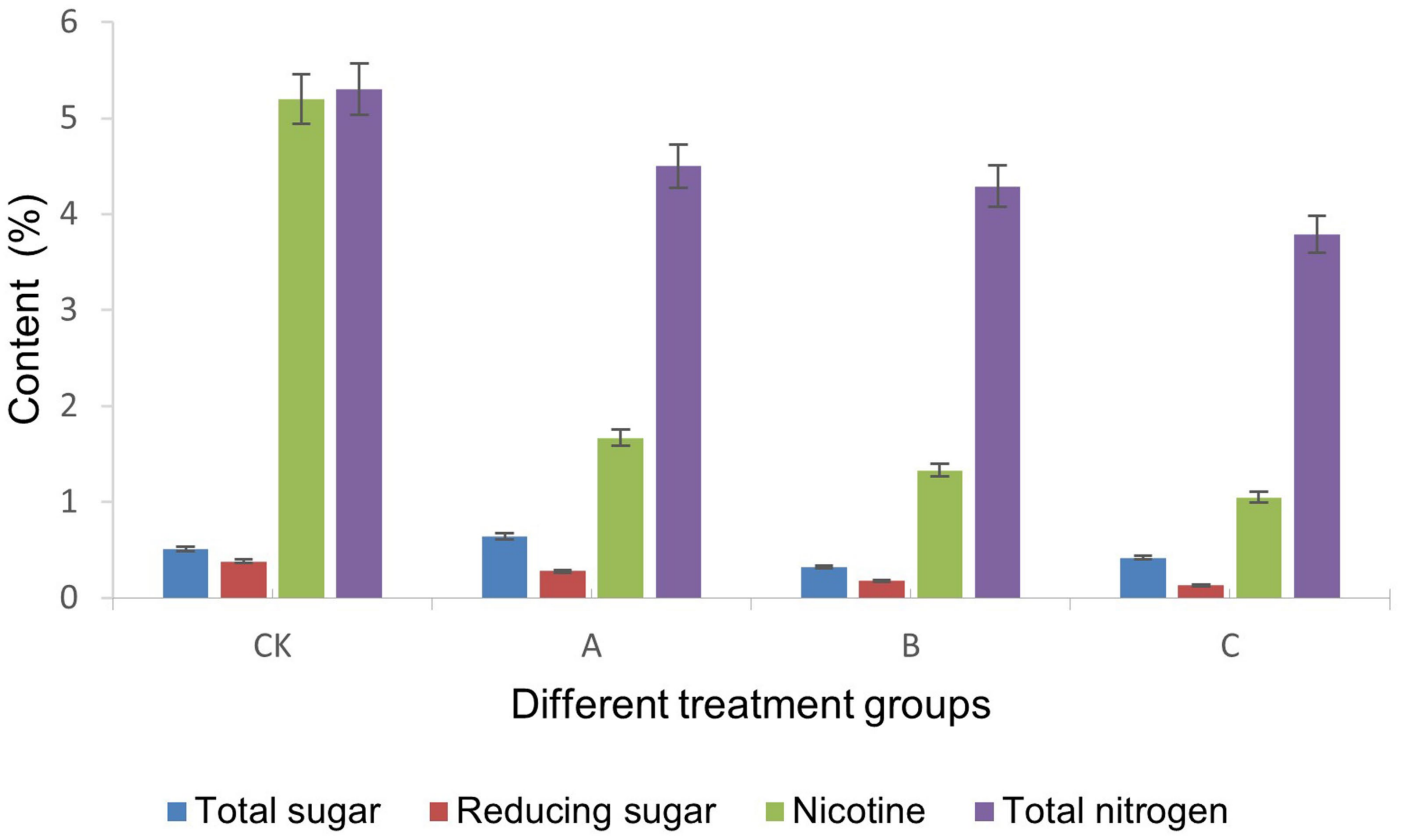
The content of conventional chemical components in four cigar tobacco leaves.

The nutrient composition serves as the main abiotic factor influencing the composition and structure of the microbial community (Molnar et al., 2025). The total sugar, reducing sugar, nicotine, and total nitrogen in cigar tobacco leaves were the main nutrients affect the growth of microbial communities. The content of total sugar, reducing sugar, nicotine, and total nitrogen in cigar tobacco leaves were determined as shown in Figure 2.

The results showed that the content of total nitrogen in CK CTLs was slightly higher than those in the high-quality CTLs (A/B/C), and the most significant difference was the nicotine content: the nicotine content in CK CTLs was 5.26 ± 0.15%, which was significantly higher than that in A (1.67 ± 0.09%), B (1.33 ± 0.08%) and C (1.05 ± 0.10%) (*p* < 0.01). Nicotine has a strong toxic effect on microbial growth. It can destroy the structure and function of the cell membrane, affect the respiration-related enzyme system, and interfere with gene expression regulation (Deng et al., 2025). Samples with lower nicotine levels may provide a more favorable environment for the growth of certain microorganisms.

### 3.2 Analysis of bacterial communities

**Figure 3:**
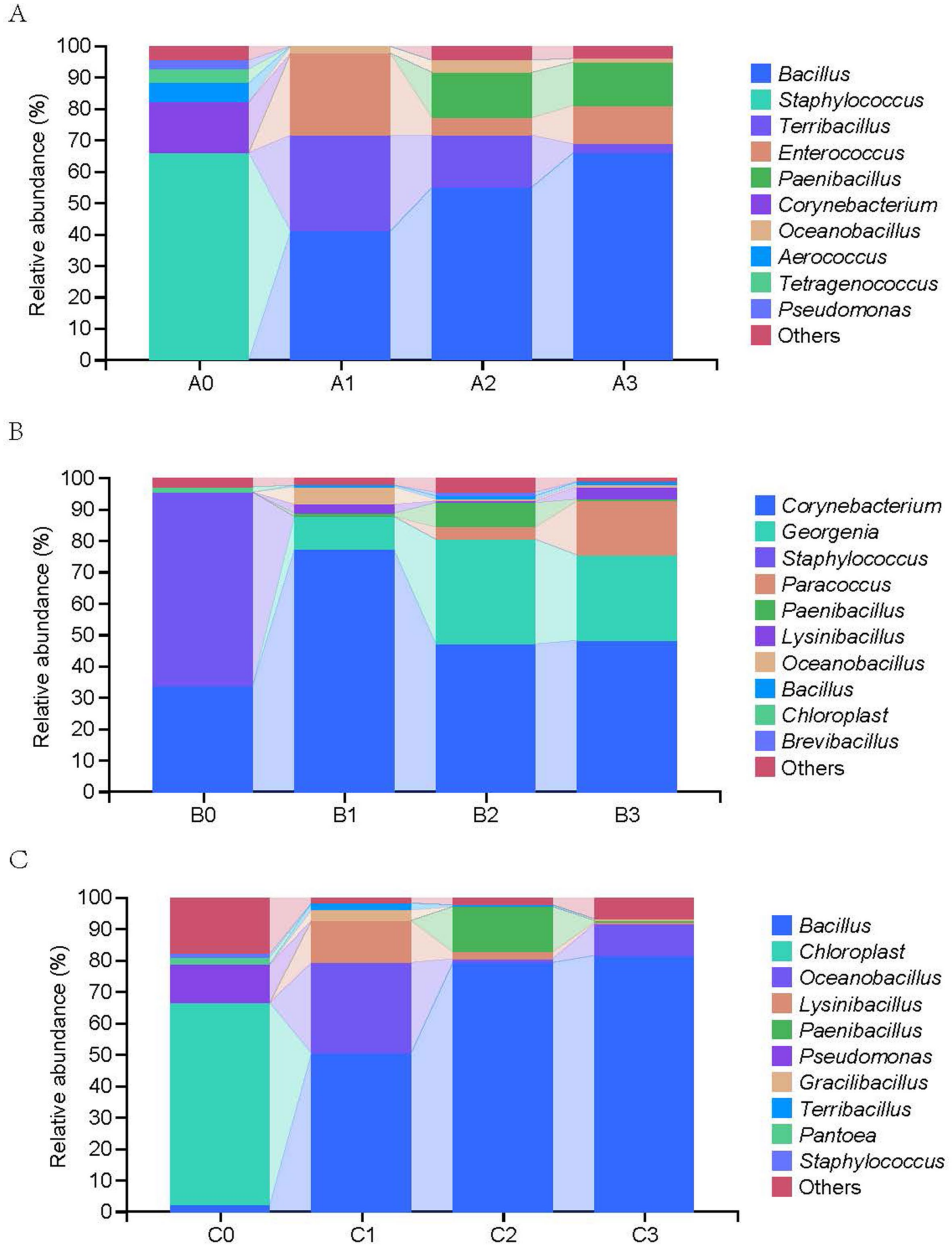
Succession of dominant bacterial communities during the enrichment and subculturing of consortia **(A-C)**. Relative abundance (%) of the top 10 dominant bacterial genera in consortia **(A-C)** across three successive subculturing generations (0: initial natural community, 1–3: subcultured generations).

High-throughput sequencing of 36 samples (12 groups × 3 biological replicates) generated 817,169 sequence reads. After quality control processes including filtering, denoising, merging, and removing non-chimeric sequences, approximately 704,647 high-quality sequences were obtained, with an average of 117,441 sequences per sample. The rarefaction depth for all sequencing samples was uniformly set to 30,000 sequences per sample, and the rarefaction curves of all samples reached saturation; the average sequencing coverage of all samples was 99.2 ± 0.3%, indicating that the sequencing data can well represent the actual microbial community structure of the samples. Taxonomic analysis showed significant changes in the bacterial community composition during the three rounds of subculture (Figure 3). The initial dominant genera of A0 were *Staphylococcus* (66.2% ± 2.3%) and *Corynebacterium* (16.1% ± 1.9%), and after three rounds of subculture, the community was dominated by *Bacillus* (66.2% ± 2.7%) and *Paenibacillus* (13.5% ± 1.3%). The initial dominant genera of B0 were *Staphylococcus* (61.4% ± 2.3%) and *Corynebacterium* (34.0% ± 1.9%), and after three rounds of subculture, the community was dominated by *Corynebacterium* (48.4% ± 2.8%) and *Georgenia* (27.1% ± 1.7%). the initial dominant genera of C0 were *chloroplast* (64.2% ± 2.7%) and *Pseudomonas* (12.1% ± 1.7%), and after three rounds of subculture, the community was dominated by *Bacillus* (81.5% ± 1.5%), with other genera accounting for less than 5%.

**Figure 4:**
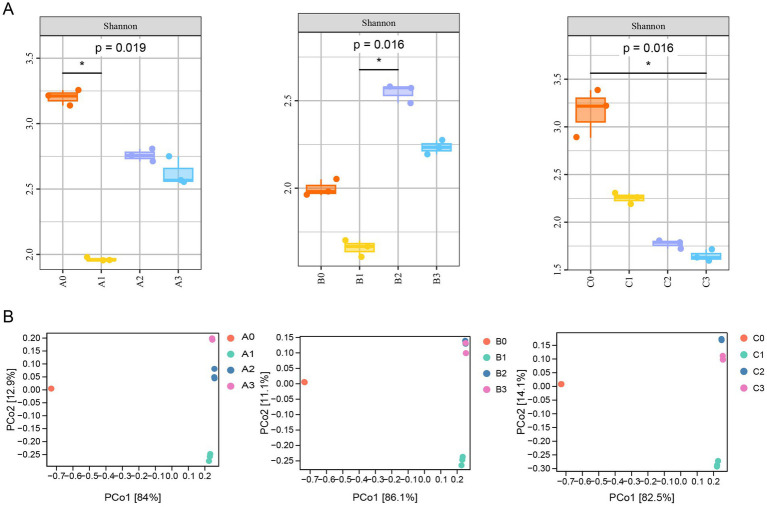
Changes in *α*-diversity **(A)** and β-diversity **(B)** of microbiotas during the construction of enriched stable consortia with short-term laboratory stability.

The Shannon index was used to evaluate the alpha diversity of the bacterial community. The Shannon index decreased from 4.1 ± 0.3 (A0) to 2.8 ± 0.2 (A3, *p* < 0.01, *t*-test), indicating a decrease community diversity. However, during three rounds of culturing, bacterial community A0 and B0 first decreased and then increased (Figure 4A). Furthermore, PCoA based on weighted UniFrac distances showed significant separation between initial and evolved communities (PERMANOVA, *R*^2^ = 0.62, *p* = 0.001, Figure 4B). The weighted UniFrac distance similarity between the 2nd and 3rd subcultures of A3/B3/C3 was >0.90 (*p* > 0.05), and the coefficient of variation (CV) of the relative abundance of dominant genera in the 3rd subculture and three consecutive subcultures after that was <10%. Nevertheless, there was only a slight difference between the second round and the third round among the three bacterial communities. This indicated that after two to three rounds of culturing, the structure of the bacterial community reached short-term stability under laboratory subculture conditions.

### 3.3 Changes in bacterial predicted functional potential

**Figure 5:**
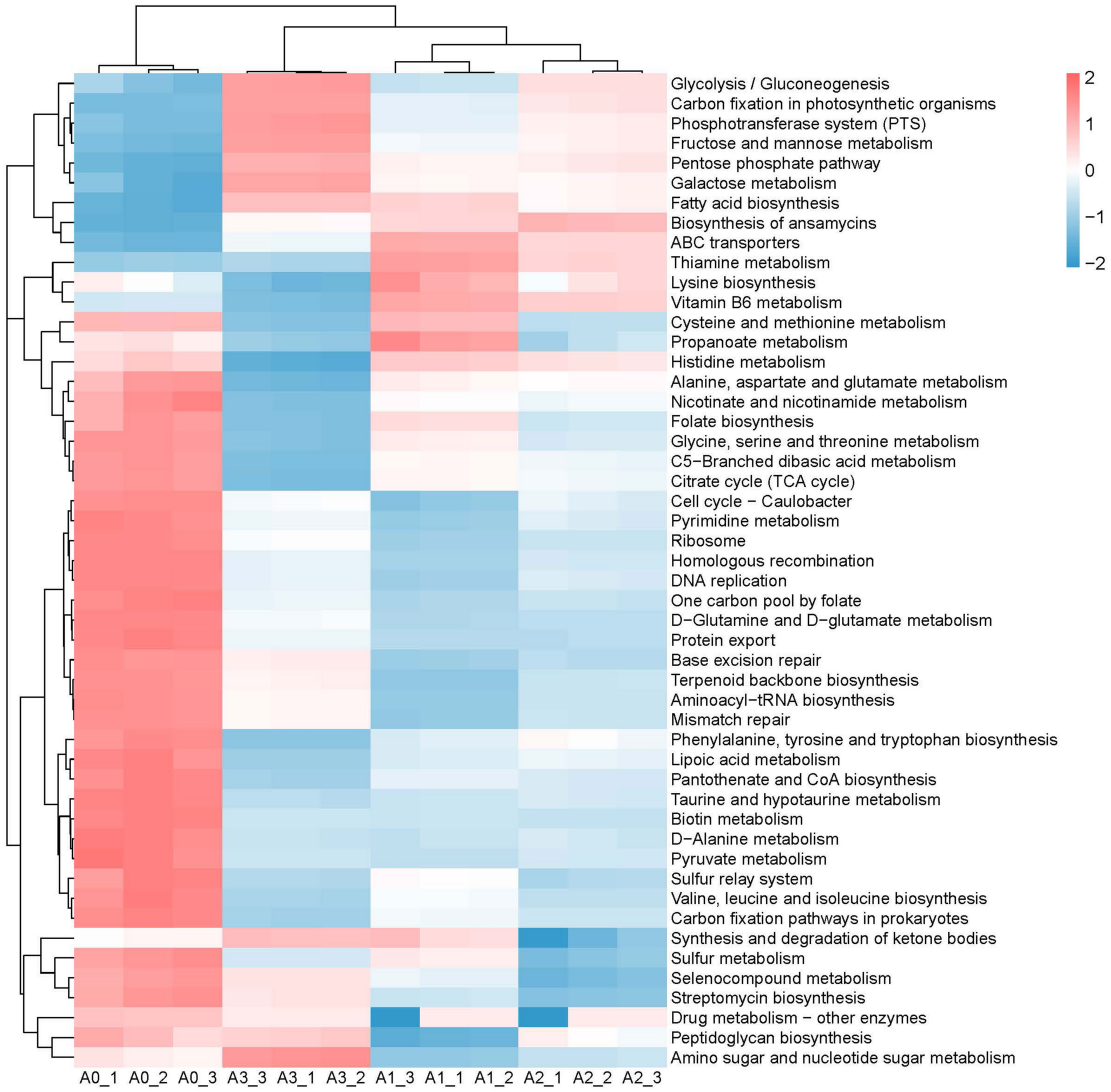
The main metabolic functions in microbiota A0, A1, A2, and A3.

**Figure 6:**
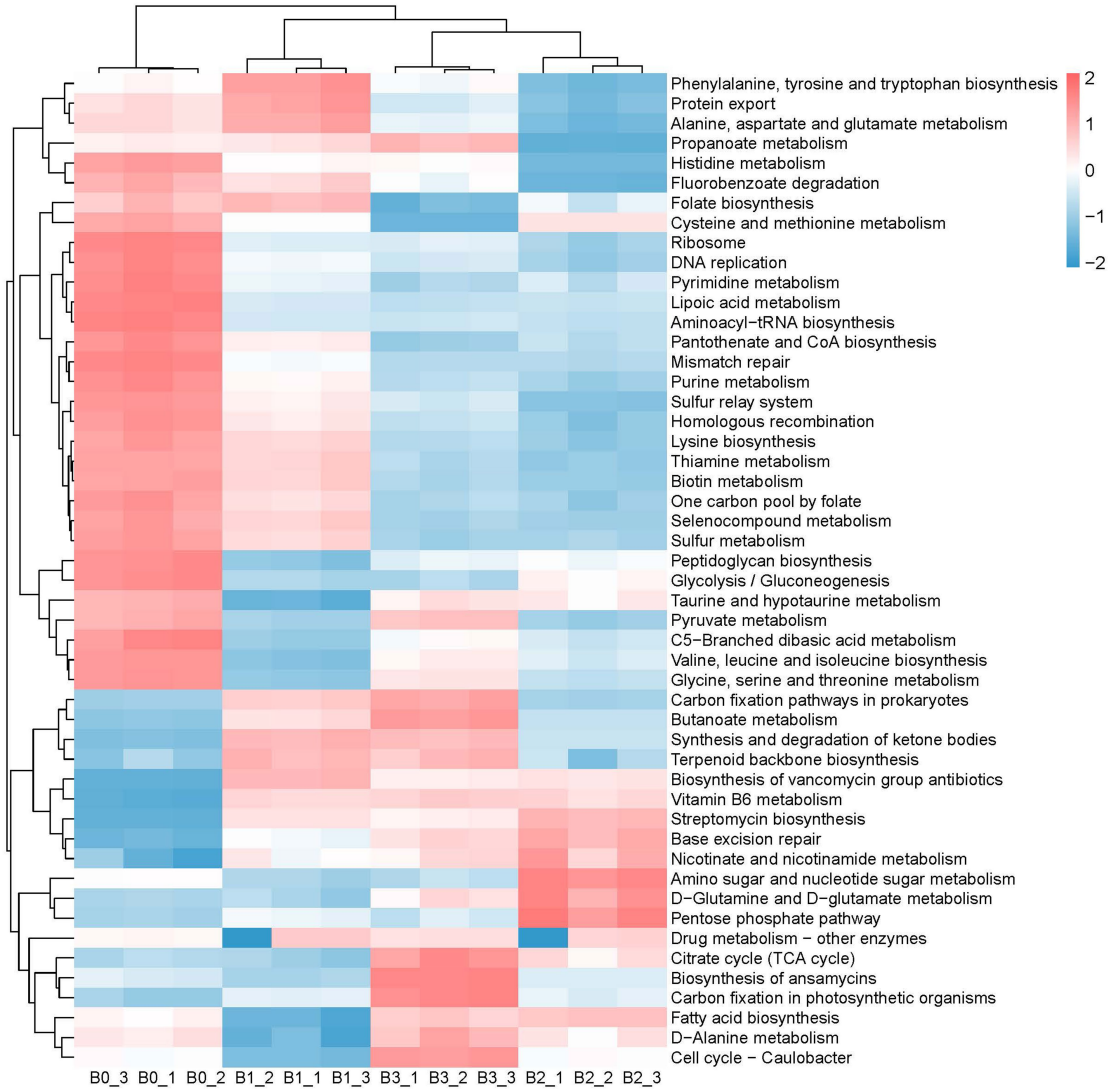
The main metabolic functions in microbiota B0, B1, B2, and B3.

**Figure 7:**
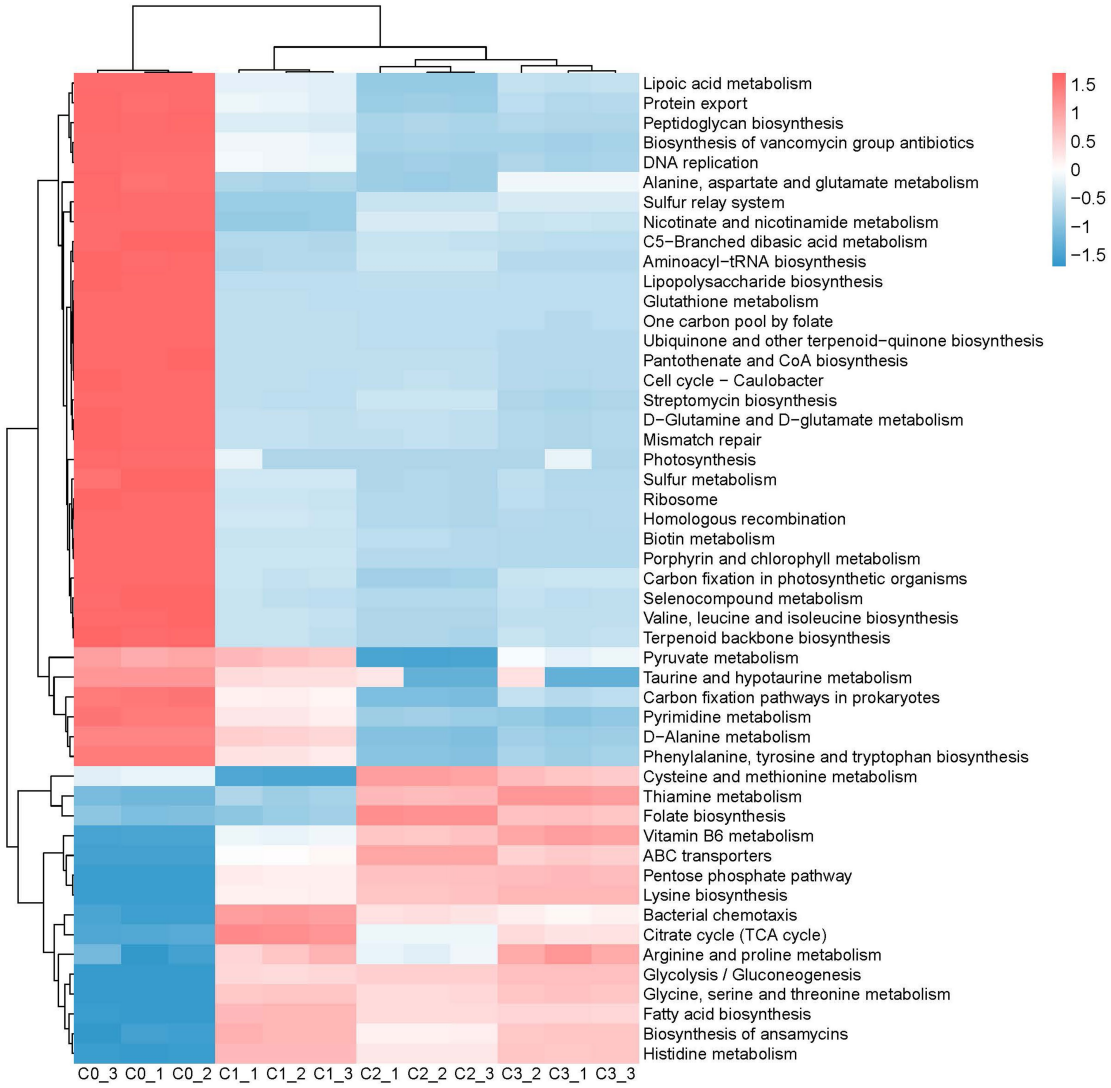
The main metabolic functions in microbiota C0, C1, C2, and C3.

The changes in the composition and structure of bacterial communities naturally affect their functional potential. The metabolic functional potential of the bacterial community was predicted by using picrust2, and the functional changes between the natural bacterial communities and the constructed enriched consortia were visually displayed by heatmap. As depicted in Figures 5–7, after three rounds of culturing, the abundance of a large number of bacterial metabolic pathways decreased significantly, perhaps because the microbial community primarily abandons most of its metabolic functional potential in order to better adapt to the new growth environment. The predicted functional potential of the enriched consortia was mostly related to the metabolism of sugars, amino acids and fatty acids. For instance, metabolic pathways related to the synthesis and degradation of ketone bodies, fructose and mannose metabolism, pentose phosphate pathway, galactose metabolism, and fatty acid biosynthesis were significantly enriched in bacterial community A3. In bacterial community B3, fatty acid biosynthesis, D-alanine metabolism, streptomycin biosynthesis, and nicotinate and nicotinamide metabolism were also enriched. In bacterial community C3, cysteine and methionine metabolism, thiamine metabolism, folate biosynthesis, vitamin B6 metabolism, pentose phosphate pathway, lysine biosynthesis, arginine and proline metabolism, glycolysis/gluconeogenesis, glycine, serine and threonine metabolism, fatty acid biosynthesis, and histidine metabolism were significantly enriched. The predicted functional potential of the enriched consortia was concentrated on sugar, amino acid and fatty acid metabolism, suggesting that it may realize colonization and functional play in the cigar tobacco leaf environment through metabolic adaptation. In addition, the consortia cultured in the second and third rounds showed consistency in enriched metabolic functional potential, which also proves that the constructed enriched consortia tend to achieve short-term functional stability in laboratory culture. It should be noted that the functional analysis in this study was based on PICRUSt2 prediction of 16S rRNA gene sequencing data, which only reflects the potential functional profile of the microbial community and cannot represent the actual metabolic activity. The actual functional expression of the consortia needs to be further verified by metagenomics, metatranscriptomics, metabolomics, and enzyme activity assays.

### 3.4 Microbial interactions

**Figure 8:**
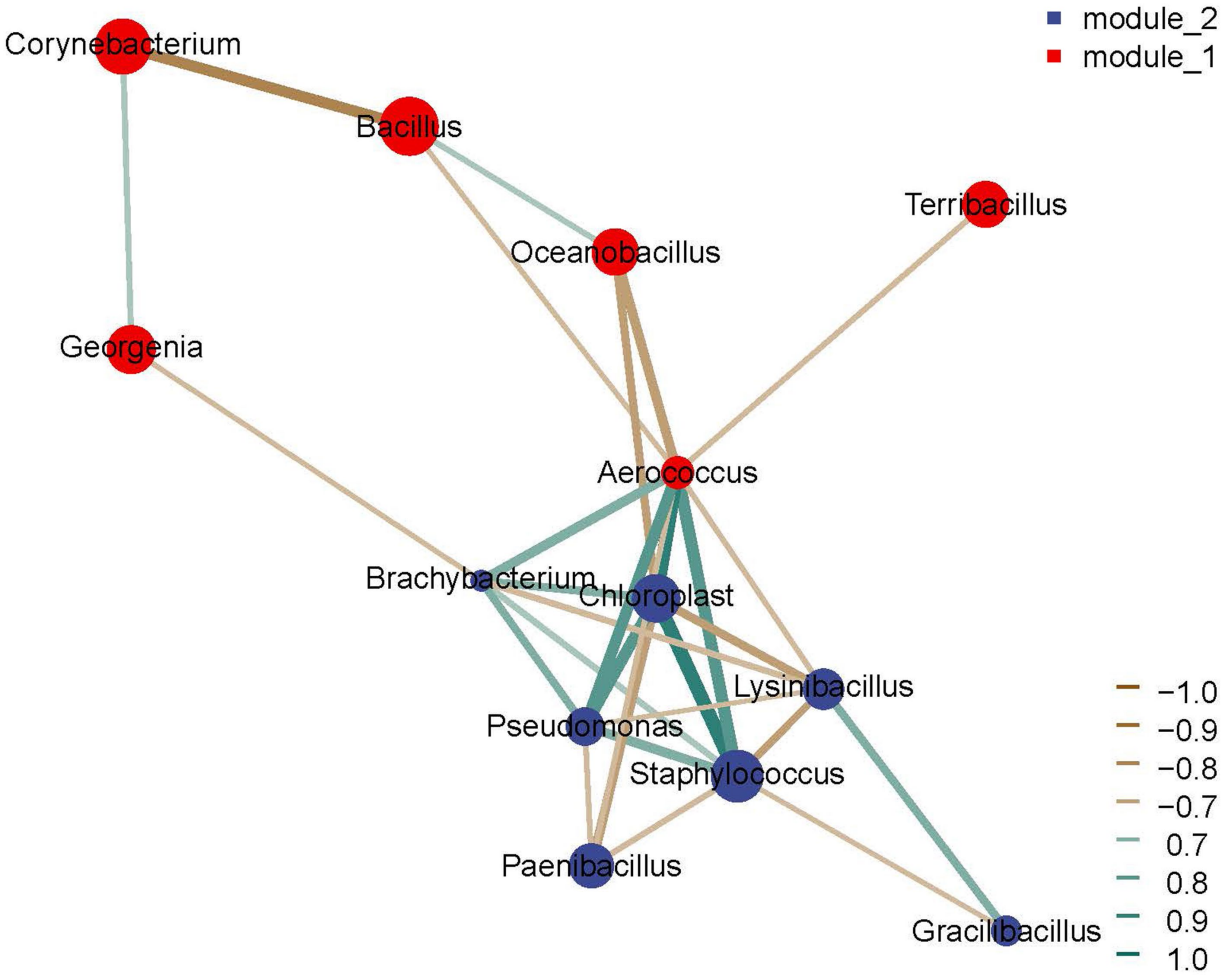
The interactions between dominant microorganisms during the construction of enriched stable consortia with short-term laboratory stability.

Microbial interactions are the core biotic factors driving the succession of enriched consortia for cigar tobacco leaves (Liang et al., 2026). Spearman correlation analysis and co-occurrence network analysis were performed to explore the microbial interactions within the enriched consortia (Wen et al., 2025), and the results showed complex positive and negative correlations between dominant genera (Figure 8). *Corynebacterium* showed a positive correlation with *Georgenia*, but a negative correlation with *Bacillus* and *Aerococcus*. *Aerococcus* had a positive correlation with *Pseudomonas*, *Brachybacterium*, *Staphylococcus*, and *Chloroplast*, and a negative correlation with *Terribacillus*. Different bacterial members interact with each other in a complex way. The positive correlations between *Corynebacterium* and *Georgenia* may indicate a cooperative relationship, possibly in resource utilization or metabolic processes. In contrast, the negative correlations between *Corynebacterium* and *Bacillus* or *Aerococcus* could imply competitive interactions, where these bacteria may be competing for the same ecological niches or resources. Similarly, the positive correlations of *Aerococcus* with several other bacterial groups might suggest synergistic interactions that enhance their survival and growth in specific growth conditions. The negative correlation with *Terribacillus* could potentially indicate antagonistic interactions, where *Aerococcus* might suppress *Terribacillus*. These complex microbial interactions played a crucial role in driving the succession and stabilization of the bacterial community during the enrichment process.

### 3.5 Sensory evaluation of fermented cigar tobacco leaves

Sensory evaluation results showed that the fermentation of CTLs with the enriched consortia significantly improved their sensory quality (Table 1). One-way ANOVA combined with Tukey HSD *post hoc* test showed that the total sensory scores of the inoculated groups (A3/B3/C3) were significantly higher than those of the control group (CK-S) (*p* < 0.01). Key sensory improvements included aroma quality, where the scores of A3/B3/C3 were 24.1 ± 1.1, 25.1 ± 1.0, and 25.9 ± 0.9, respectively, 25.6–31.1% higher than that of CK-S (19.2 ± 1.3); aroma intensity, with scores of A3/B3/C3 at 15.7 ± 0.7, 16.5 ± 0.6, and 17.2 ± 0.5, 36.5–49.6% higher than that of CK-S (11.5 ± 0.9); irritation, where the scores of A3/B3/C3 were 8.1 ± 0.6, 8.5 ± 0.8, and 8.3 ± 0.7, showing no significant difference compared with CK-S (8.2 ± 0.7) (*p* > 0.05); aroma richness, with scores of A3/B3/C3 at 13.2 ± 0.9, 13.9 ± 0.8, and 14.0 ± 0.8, 30.2–38.5% higher than that of CK-S (10.1 ± 0.8); and sweetness, where the scores of A3/B3/C3 were 10.4 ± 0.5, 11.1 ± 0.4, and 11.1 ± 0.4, 42.3–51.8% higher than that of CK-S (7.3 ± 0.6). Cohen’s d effect size analysis showed that the effect sizes of A3, B3, and C3 on the total sensory score were 2.13, 2.45, and 2.67, respectively (all Cohen’s *d* > 0.8), indicating a large effect size of the enriched consortia in the association with improved sensory quality of CTLs, and these results clearly demonstrated that the quality improvement of CTLs was closely associated with the biological activity of the enriched stable consortia.

**Table 1:** Sensory scores of cigar tobacco leaves in different groups (mean ± SD, 100-point system).

Group	Aroma quality (30)	Aroma intensity (20)	Irritation (15)	Aroma richness (20)	Sweetness (15)	Total score (100)
CK-S	19.2 ± 1.3	11.5 ± 0.9	8.2 ± 0.7	10.1 ± 0.8	7.3 ± 0.6	56.3 ± 3.1
A3	24.1 ± 1.1**	15.7 ± 0.7**	8.1 ± 0.6	13.2 ± 0.9**	10.4 ± 0.5**	68.6 ± 2.7**
B3	25.1 ± 1.0**	16.5 ± 0.6**	8.5 ± 0.8	13.9 ± 0.8**	11.1 ± 0.4**	71.5 ± 2.4**
C3	25.9 ± 0.9**	17.2 ± 0.5**	8.3 ± 0.7	14.0 ± 0.8**	11.1 ± 0.4**	72.9 ± 2.2**

** indicates extremely significant difference compared with CK-S (*p* < 0.01) after Benjamini–Hochberg FDR correction; data are the average of 3 independent sensory evaluation repetitions.

## 4 Discussion

In recent years, the cigar market in China has expanded rapidly; however, the quality of domestically produced CTLs remains generally suboptimal due to underdeveloped fermentation technologies (Zheng et al., 2022a,b). Key issues include unbalanced internal chemical compositions, inadequate aroma complexity, and pronounced irritation, which hinder their industrial applicability. Optimizing the microbial community during fermentation has emerged as a viable strategy to enhance CTL quality (Wang et al., 2024), but the natural microbial community of CTLs is randomly derived from the environment, leading to unstable fermentation quality (Ren et al., 2023). This study constructed enriched stable consortia with short-term laboratory stability for CTL fermentation via the top-down approach.

Unlike single-strain inoculation (Liu et al., 2025; Yao et al., 2025), the enriched consortia retained the natural synergistic interactions between microorganisms, which enhanced their environmental adaptability and fermentation efficiency. In addition, compared with bottom-up synthetic consortia, it has the advantage of making full use of the functional diversity and synergistic effects of natural microbial communities without in-depth understanding of the interaction mechanisms within the consortium, enabling the rapid acquisition of synthetic microbiotas with complex functions, which is suitable for solving some practical application problems.

The sequencing results prove that the constructed bacteria community maintained relatively short-term stability under laboratory subculture conditions after undergoing three rounds of culture. In the constructed bacteria community, the dominant genera were *Bacillus*, *Corynebacterium,* and *Georgia*. Existing tobacco fermentation studies have shown that *Bacillus* can improve tobacco quality by degrading starch and protein and produce flavor precursors. For example, Wen et al. (2023) added *Bacillus Gauss* with protease-producing ability to the tobacco leaves during fermentation, which led to a reduction in irritation and impurities and an increase in the aroma of the tobacco leaves. Ma et al. (2024) screened a *Bacillus subtilis* with the simultaneous degradation ability of starch (with a degradation rate of 33.87%) and protein (with a degradation rate of 20%) to improve the quality of low-class tobacco leaf. *Corynebacterium* participates in nitrogen metabolism and synthesizes aroma compounds such as aldehydes and ketones (Zheng et al., 2022a,b). *Georgenia* is reported for the first time to be enriched in CTL fermentation, with its specific functions needing further verification by pure strain isolation and functional validation, its positive correlation with *Corynebacterium* suggests potential cooperative interactions that may contribute to the observed sensory improvements. Collectively, the functional traits of these dominant genera and their synergistic metabolic activities form the basis for the comprehensive sensory quality enhancement of CTLs that is associated with the enriched consortia.

A critical consideration for the industrial application of microbial consortia is their stability, and this study only verified that the enriched consortia had short-term community structure stability under laboratory-scale subculture conditions (Bray–Curtis dissimilarity < 0.1, weighted UniFrac similarity > 0.90) and short-term fermentation functional stability in laboratory fermentation trials (consistent sensory improvement after 3 consecutive subcultures). Notably, the current fermentation validation experiment has a key limitation in its experimental design for causal inference: sterilized substrate and heat-killed inoculum controls were not included, which restricts the ability to definitively attribute the observed sensory quality improvements solely to the biological activity of the enriched consortia. The exclusion of these controls was based on the goal of simulating the actual practical production conditions of CTL fermentation, where sterile substrates and inactivated microbial inocula are not applied, and this practical consideration formed the rationale for the experimental design. However, this deficiency means that the observed effects cannot be fully disentangled from potential abiotic factors or indirect microbial influences, and the quality improvement of CTLs can only be stated as being closely associated with the inoculation of the enriched consortia, rather than directly caused by their biological activity.

In addition to the above experimental design limitation, it should be further emphasized that the short-term stability verified in this study is far from meeting the requirements of industrial application, and key industrial application stability indicators such as long-term storage stability, batch-to-batch fermentation consistency, and resistance to exogenous microbial contamination still lack any validation data. Future research will focus on optimizing the stability of the consortia for industrial application, including evaluating the activity of the consortia under different storage conditions (4 °C refrigeration, −20 °C freezing, freeze-drying) to screen the optimal storage method, verifying the community structure and fermentation function of the consortia after 10 consecutive subcultures to assess long-term subculture stability, testing the ability of the consortia to resist exogenous contaminant, and conducting 3 consecutive batches of pilot-scale fermentation (10 kg CTLs per batch) to verify the consistency of the fermentation effect.

The fermentation results have demonstrated that constructed consortia with dominant species can attain excellent fermentation properties in laboratory trials, and their inoculation is associated with significant improvements in CTL sensory quality. Aroma quality scores of the inoculated groups (A3/B3/C3) increased by 25.6–31.1% compared to CK-S, aroma intensity by 36.5–49.6%, and aroma richness by 30.2–38.5%, reflecting enhanced purity, concentration, and layering of CTL aroma.

In summary, this study applied the method of microbial consortium engineering to cigar tobacco leaf fermentation, established a technical route of “natural microbial community – directional enrichment – consortium with short-term laboratory stability – fermentation application”, and provided a new idea for the microbial regulation of tobacco fermentation. At the same time, it enriched the research content of fermentation microorganisms for cigar tobacco leaves, clarified the dominant genera and their interactions, and laid a foundation for the subsequent screening of core functional strains and the construction of synthetic microbial consortia. However, this study also has several specific limitations that need to be addressed in future research. First, functional analysis was based on PICRUSt2 prediction, and actual metabolic activity of the consortia requires verification through multi-omics approaches (metagenomics, metatranscriptomics, and metabolomics) and enzyme activity assays (e.g., amylase, fatty acid synthase, and nicotine dehydrogenase). Second, core functional strains in the consortia have not been isolated and identified, and their individual and synergistic effects need to be validated through strain reconstitution. Third, in-depth research is still needed to fully understand the specific mechanisms by which the constructed bacterial community is associated with CTL quality improvement, including identifying key functional strains and their interaction networks. Fourth, the lack of sterilized substrate and heat-killed inoculum controls in fermentation validation limits causal inference, and this will be rectified in future studies. In practical CTL fermentation, the synthetic microbial community also requires rational control of fermentation conditions (temperature, humidity, and time) to promote microbial activity and achieve optimal fermentation outcomes. Precise regulation of these parameters will ensure consistent fermentation processes across batches, leading to stable high-quality CTLs. Future research will integrate top-down and bottom-up approaches, isolating core functional strains from the enriched consortia to construct defined synthetic microbial communities, and optimizing their fermentation performance through environmental regulation. This integrated strategy will combine the advantages of natural consortia (strong adaptability) and synthetic consortia (precise regulation), providing a more efficient and stable microbial solution for CTL fermentation.

## 5 Conclusion

This study constructed enriched stable consortia with short-term laboratory stability for CTL fermentation via the top-down approach using natural microbial communities from high-quality CTLs. The constructed consortia were dominated by *Bacillus*, *Corynebacterium*, and *Georgenia*. Fermentation with the consortia significantly improved the sensory quality of CTLs (large effect size, Cohen’s *d* > 0.8), including enhanced aroma quality, aroma intensity, sweetness, and aroma richness. The method for constructing enriched stable consortia proposed in this study is simple, efficient, and suitable for practical production. It makes full use of natural microbial resources and provides a microbiological solution to solve the problem of unstable natural fermentation of CTLs. Future research will focus on verifying the industrial application stability of the consortia, clarifying their functional mechanism, and isolating core functional strains for synthetic community construction.

## Data Availability

The original contributions presented in the study are publicly available. This data can be found here: NCBI (https://www.ncbi.nlm.nih.gov/bioproject) accession number PRJNA1203956.
